# A Novel Method for Predicting Recirculation by Sweep-Gas Control in Extracorporeal Membrane Oxygenation

**DOI:** 10.5761/atcs.oa.26-00027

**Published:** 2026-07-16

**Authors:** Tomoki Tahara, Daisuke Sakota, Nobutomo Morita, Ryo Kosaka, Katsuhiro Ohuchi, Tetsuhito Kigata, Yusuke Tsuboko, Tomoyuki Fujita, Eiki Nagaoka

**Affiliations:** 1 Department of Cardiovascular Surgery, Graduate School of Medical and Dental Sciences, Institute of Science Tokyo, Tokyo, Japan; 2 Health and Medical Research Institute, National Institute of Advanced Industrial Science and Technology (AIST), Tsukuba, Ibaraki, Japan; 3 Sensing System Research Center, National Institute of Advanced Industrial Science and Technology (AIST), Tosu, Saga, Japan; 4 Department of Clinical Engineering, Faculty of Medical Science, Juntendo University, Tokyo, Japan; 5 Department of Anatomy and Neurobiology, National Defense Medical College, Tokorozawa, Saitama, Japan; 6 Laboratory of Veterinary Anatomy, Tokyo University of Agriculture and Technology, Fuchu, Tokyo, Japan; 7 Division of Medical Devices, National Institute of Health Sciences, Kawasaki, Kanagawa, Japan

**Keywords:** extracorporeal membrane oxygenation, recirculation, cardiopulmonary bypass, hemodynamics, medical devices

## Abstract

**Purpose:**

Recirculation is a critical issue in veno-venous extracorporeal membrane oxygenation (VV ECMO). We propose a novel method for estimating the recirculation ratio using temporary sweep-gas control.

**Methods:**

A circuit simulating VV ECMO recirculation was constructed by introducing a short circuit between the inlet and outlet of the oxygenator, enabling direct measurement of recirculation flow. Temporary cessation of sweep gas induced decreases in oxygen saturation at the oxygenator inlet (S_pre_O_2_) and outlet (S_post_O_2_). The ratio of their temporal changes (⊿S_pre_O_2_/⊿S_post_O_2_) converged to the recirculation ratio, reflecting blood mixing. The early prediction phase was defined as the interval required for oxygen saturation to decrease from 99% to 95% after sweep-gas cessation. The method was validated in an acute porcine model.

**Results:**

In circuit experiments, the relative prediction error was 7.0% ± 5.2%. Early prediction required 3.0 ± 1.0 s when recirculation was ≤50% and 4.1 ± 1.0 s when >50%. In animal experiments, the error was 7.5% ± 4.0%, with times of 10.4 ± 2.8 s for recirculation ≤50% and 27.8 ± 0.5 s for >50%.

**Conclusions:**

The proposed method enables rapid, less invasive prediction of the recirculation ratio and may provide a practical tool for optimizing ECMO management.

## Introduction

Extracorporeal membrane oxygenation (ECMO) became widely recognized during the 2009 influenza A (H1N1) pandemic^1)^ and was again highlighted during the COVID-19 pandemic.^2,3)^ Veno-venous ECMO (VV ECMO) plays a critical role in the management of patients with severe hypoxemic respiratory failure.^4)^ However, due to the inflow and outflow structure of VV ECMO, a portion of the oxygenated blood can be continuously drained back into the circuit, reducing oxygen delivery to the patient’s systemic circulation. This phenomenon is known as recirculation.^5)^

Recirculation in VV ECMO is affected by multiple factors, including cannula configuration and positioning, pump speed, cannula size, extracorporeal blood flow, and variations in intrathoracic or intracardiac pressure.^6,7)^ In clinical settings, when systemic oxygenation becomes insufficient, clinicians often increase ECMO blood flow to enhance gas exchange. However, excessive pump flow can lead to negative venous pressure at the drainage cannula, resulting in circuit chattering and inadequate venous drainage. In such situations, interventions such as volume supplementation, cannula repositioning, or exchange to a different cannula type, including a bicaval dual-lumen cannula, may be required.^7–9)^ Although dual-lumen cannulas have been reported to reduce recirculation compared with conventional dual-site configurations, their performance remains influenced by flow dynamics and patient anatomy, their use may be limited by body size, and they may increase the risk of hemolysis or thrombosis due to high shear stress and flow stagnation.^7,10–13)^

Continuous quantitative monitoring of recirculation is not routinely available in VV ECMO. In clinical practice, recirculation is therefore inferred from indirect indicators such as inadequate oxygenation, a limited increase in oxygen delivery despite higher ECMO flow, and hemolysis. To enable more objective assessment, several quantitative methods have been developed, including the ultrasound dilution technique (UDT), the step-change technique based on sweep-gas modulation, and the mixed venous oxygen saturation (SvO_2_) method.^6,7,10,11,14–20)^ Among these, UDT provides a reliable and quantitative assessment by analyzing ultrasound velocity changes in the ECMO circuit following the injection of a small saline bolus and is considered the current standard. The UDT-based ELSA Monitor (Transonic Systems, Ithaca, NY, USA) allows bedside measurement and has been introduced in several regions, though it is not yet widely available.^10,11,17,21)^ Another quantitative approach is the step-change technique, which estimates recirculation by analyzing oxygenation responses following abrupt sweep-gas modulation. However, it requires intentional circuit perturbation and time-dependent curve fitting, which may limit its suitability for rapid or continuous bedside assessment.^20)^ The SvO_2_ method has long been regarded as the standard for comparison, but it requires turning off the ECMO sweep gas for a long time and is therefore not always feasible in clinical practice.

In this study, we propose a novel method for predicting recirculation fraction by briefly turning off the sweep gas and using simultaneous real-time optical monitoring. This approach allows for immediate prediction, requires no invasive blood sampling, and can be easily performed at the bedside within a short period of time. Accurate assessment of recirculation allows for the optimization of ECMO settings, which may, in turn, reduce unnecessary fluid administration and medication, as well as potentially facilitate earlier ECMO weaning. The validity of the proposed method was evaluated through in vitro circuit simulations and in vivo animal experiments.

## Materials and Methods

### In vitro ECMO recirculation model

**Fig. 1:**
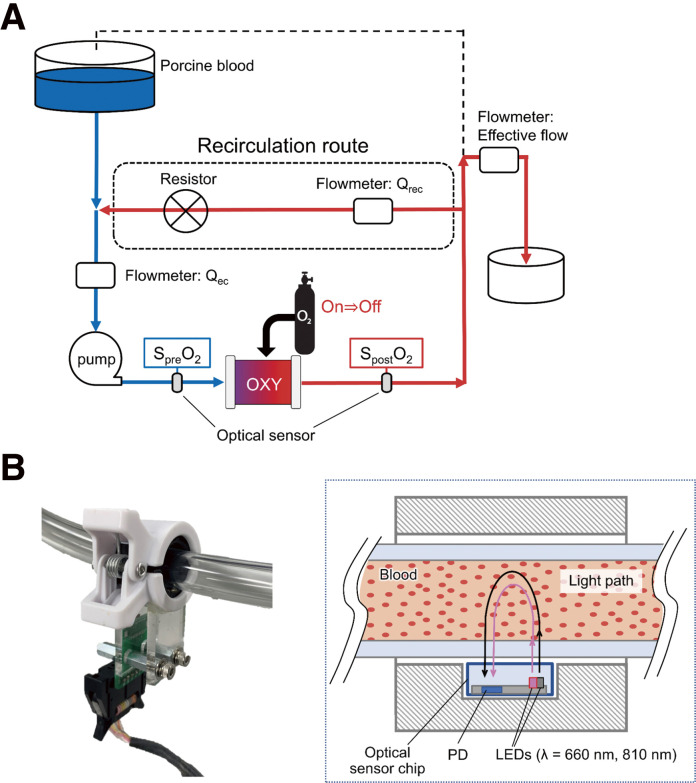
Setup of an in vitro ECMO recirculation model. (**A**) Section schematic of the circuit. Porcine blood deoxygenated to approximately 70% saturation in the upper reservoir simulates venous saturation. A recirculation route connects oxygenated and deoxygenated blood, and the recirculated volume was controlled using a resistor. Blood flowing from the red line to the blue line through the recirculation route represents the recirculation fraction (Q_rec_). (**B**) Optical sensor for real-time SbO_2_ monitoring. The optical sensor chip is embedded in the inner wall of the clamp. The optical sensor chip can detect reflected light intensity from blood at the wavelengths of 660 nm (red) and 810 nm (near-infrared). SbO_2_ can be estimated from the ratio of these light intensities. ECMO, extracorporeal membrane oxygenation; Q_rec_, recirculation flow; SbO_2_, blood oxygen saturation; S_pre_O_2_, inlet of the oxygenator; S_post_O_2_, outlet of the oxygenator; PD, photodiode; LED, light-emitting diode

Fresh porcine blood was obtained from a local slaughterhouse (TOKYOSHIBAURAZOUKI Co., Ltd., Tokyo, Japan) and anticoagulated with 3.2% sodium citrate at a ratio of 1 part citrate to 9 parts blood. The blood was circulated through a custom ECMO recirculation model comprising 2 reservoirs, a blood pump (MERA; SENKO Medical Instrument Mfg. Co., Ltd., Tokyo, Japan), polyvinyl chloride tubing, a membrane oxygenator (BioCube 6000 P; Nipro Co., Ltd., Osaka, Japan), connectors, and a resistor to adjust the recirculation flow rate (**Fig. 1A**). The oxygenator has a membrane surface area of 1.3 m^2^ and an internal priming volume of 250 mL. The total priming volume of the circuit was 4 L.

Three ultrasound flowmeters (HT310; Transonic Systems Inc., Ithaca, NY, USA) were placed to measure the ECMO flow (Q_ec_), recirculation flow (Q_rec_), and effective flow (Q_eff_). Blood oxygen saturation (SbO_2_) was continuously monitored using custom-developed optical sensor probes^22)^ (**Fig. 1B**), calibrated against a blood gas analyzer (ABL80-FLEX system; Radiometer Ind., Tokyo, Japan), and placed at the inlet and outlet of the oxygenator (S_pre_O_2_ and S_post_O_2_, respectively). The mean coefficient of determination (R^2^) for these calibration curves was 0.94 ± 0.016. The temporal resolution of the oximeter system was 0.1 s.

Before each run, venous saturation was adjusted to 70% by deoxygenating the priming blood with a 93% N_2_/7% CO_2_ gas mixture. The sweep gas was then switched to 100% O_2_ (2 L/min), and the pump was initiated at 1 L/min. Blood was oxygenated in the membrane, with part recirculating and the remainder delivered to the distal reservoir. Once S_pre_O_2_ was stabilized, the sweep gas was discontinued, after which both S_pre_O_2_ and S_post_O_2_ declined. Oxygen saturation was continuously monitored until the reservoir was empty. The experiment was repeated 13 times, with the recirculation fraction intentionally varied between 0% and 80% by adjusting the resistance in the recirculation pathway.

### Measurement of recirculation fraction using SbO_2_ trend after sweep gas off

Recirculation fraction (Rf) is defined as the ratio of recirculated flow to total ECMO flow as follows:

(Eq, 1) Rf=QrecQec

On the other hand, the following equation, derived from Fick’s principle, has often been used as a conventional method to determine Rf:^4–6,8,14,16)^

(Eq, 2) Rf=(SpreO2−SvO2)(SpostO2−SvO2)

In this context, the term “SvO_2_” refers to the oxygen saturation of venous blood entering the ECMO circuit during temporary sweep-gas cessation. Although widely used in the ECMO literature, this value does not represent true mixed venous saturation from the pulmonary artery but rather reflects drainage-side venous saturation under extracorporeal circulation. Because this maneuver often requires interruption of gas exchange and increased ventilatory support, its applicability in patients with severe respiratory failure is limited.

Based on this limitation, we developed a novel method to estimate Rf without direct measurement of SvO_2_ by utilizing transient changes in oxygen saturation induced by sweep-gas interruption.

Assuming that oxygen saturation at the pre-oxygenator site is determined by linear mixing of recirculated blood and systemic venous blood, the relationship can be expressed as a function of Rf. When sweep-gas flow is transiently discontinued, oxygen transfer across the membrane oxygenator is reduced, resulting in measurable changes in both pre- and post-oxygenator oxygen saturation (S_pre_O_2_ and S_post_O_2_).

If the measurement window is sufficiently short, SvO_2_ can be considered approximately constant. Under this quasi-steady assumption, subtraction of the 2 states (before and after sweep-gas interruption) eliminates the SvO_2_ term, leading to the following relationship:

(Eq, 3) Rf=ΔSpreO2ΔSpostO2

where Δ*S*_*pre*_ *O*_2_ = *S*_*pre*_ *O*_2_ – *S*ʹ_*pre*_ *O*_2_ and Δ*S*_*post*_ *O*_2_ = *S*_*post*_ *O*_2_ – *S*ʹ_*post*_ *O*_2_.

The full mathematical derivation is provided in the **Supplementary Material**.

Importantly, Eq. (3) is derived under several simplifying assumptions, including (i) quasi-steady conditions during the brief measurement period, (ii) relative stability of venous oxygen saturation, (iii) operation within the near-linear region of the oxygen dissociation curve, and (iv) linear mixing between recirculated and systemic venous blood.

These assumptions allow the transient changes in oxygen saturation to be interpreted as proportional to the recirculation fraction within a short temporal window.

Our proposed sweep-gas control technique estimates the Rf by altering the sweep-gas flow and analyzing the corresponding changes in pre- and post-oxygenator oxygen saturation (S_pre_O_2_ and S_post_O_2_).

Rf was first set to predefined levels (Set Rf) in the experiments. Since the optimal timing for accurate Rf measurement was unclear, we compared 2 estimation approaches: (1) Rf estimated in the early phase (Rf-early), calculated from SbO_2_ changes during the initial phase after turning off the sweep gas until S_post_O_2_ decreases from 99% to 95%; and (2) Rf estimated in the end phase (Rf-end), calculated from SbO_2_ changes during the final 5 s before S_pre_O_2_ and S_post_O_2_ converge. For reference, “Measured Rf,” calculated from Eq. (2) using directly measured venous saturation and following the same principle as the conventional SvO_2_ method, was also evaluated.

### Animal experiments

All animal experiments were conducted in accordance with the Principles of Laboratory Animal Care formulated by the National Society for Medical Research and the Guide for the Care and Use of Laboratory Animals prepared by the Institute of Laboratory Animal Research (ILAR), published by the National Academies Press.

The animal study was approved by the Life Science Experiment Application Committee of the Institute of Science Tokyo and the Institutional Animal Care and Use Committee of the National Institute of Advanced Industrial Science and Technology (Approval ID: A2023-129A). A total of 3 pigs were used in this study. The number of animals was kept to the minimum required to obtain scientifically valid results. The study is reported in accordance with the ARRIVE 2.0 guidelines.

**Fig. 2:**
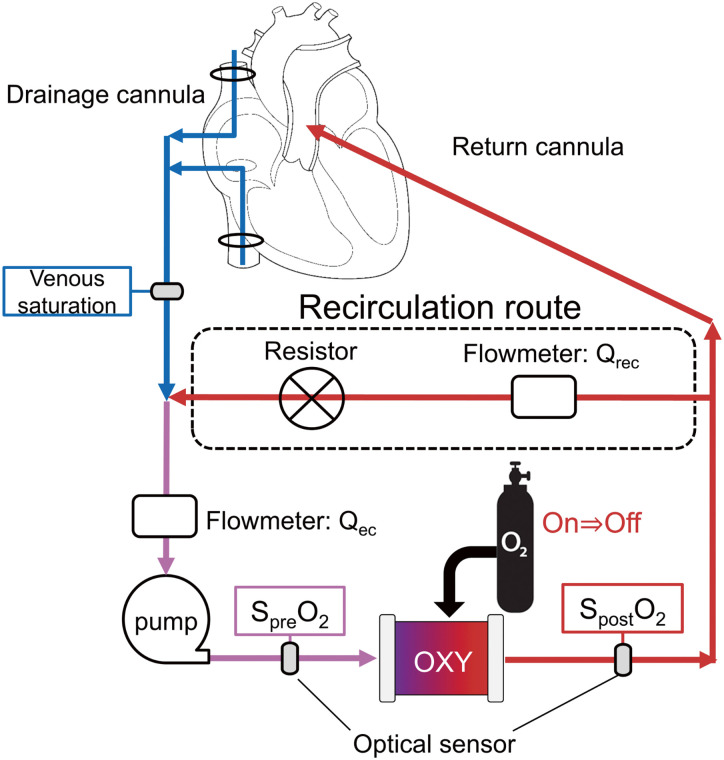
Setup of the animal experiments. A bi-caval VV ECMO configuration was established using 2 venous drainage cannulas and a pulmonary artery return cannula. A shunt between the drainage and return routes was installed to simulate recirculation. The circuit contained 1 pump with an adult-sized membrane oxygenator (BioCube 6000p; Nipro). Q_rec_, recirculation fraction; VV ECMO, veno-venous extracorporeal membrane oxygenation; S_pre_O_2_, inlet of the oxygenator; S_post_O_2_, outlet of the oxygenator

Male crossbred Landrace and Large White pigs, weighing 70 kg, were used in this study. All animals had healthy native lungs without induced injury, minimizing confounding effects of pulmonary gas exchange on oxygen saturation measurements. Tracheal intubation was performed after subcutaneous injection of 2 mg/kg xylazine and 20 mg/kg ketamine followed by inhalation of isoflurane at 1.5%–3.0%. After discontinuation of ventilation, propofol was infused as the anesthetic agent. Electrocardiogram, oxygen saturation, and arterial pressure were monitored. After heparinization, the venous drainage cannulas (32-Fr DLP Single Stage Venous Cannulae; Medtronic, Inc., MN, USA) were inserted into the superior and inferior vena cava through a median sternotomy. A return cannula (22-Fr Optisite cannula; Edwards Lifesciences, Irvine, CA, USA) was inserted into the main pulmonary artery (**Fig. 2**). During the experiment, heparin was administered as needed to maintain the activated clotting time (ACT) above 200 s. This system allows stable extracorporeal circulation while maintaining the structure of “VV ECMO” and avoiding recirculation in the right atrium. A shunt between the drainage and return routes was installed to simulate recirculation. This made it possible to precisely quantify *Rf* = *Q*_*rec*_/*Q*_*ec*_ (Eq. 1) using the flowmeters. *Q*_*ec*_ = 3 L/min; 100% O_2_ was delivered to a membrane (BioCube 6000 P; Nipro Co., Ltd.) at 3 L/min. Mechanical ventilation was discontinued during the experiment. The Set Rf was adjusted to 0%, 25%, 50%, and 75%. For each Rf, the estimation of Rf was performed 3 times. All experiments were performed under identical conditions, with the only variable being the recirculation rate. The sweep gas was turned off for only 30 s to minimize the risk of hypoxemia. Early-phase prediction was performed during this period, using the same method as in the in vitro experiment.

### Statistical analysis

All statistical analyses were performed using GraphPad Prism version 9 (GraphPad Software, San Diego, CA, USA). Linear regression and analysis of covariance were used to compare Set Rf with Rf-early and Rf-end. Agreement between methods was assessed by Bland–Altman analysis. A *p* value <0.05 was considered significant.

## Results

**Fig. 3:**
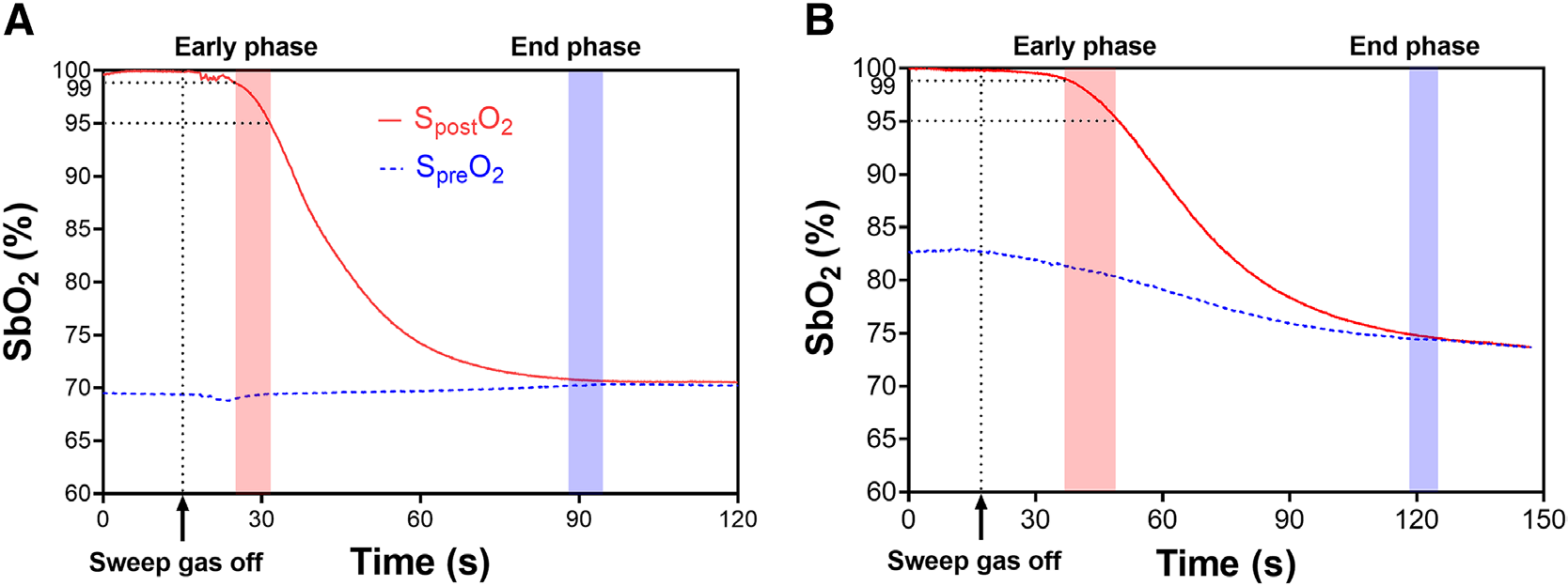
SbO_2_ changes in S_pre_O_2_ and S_post_O_2_ of the in vitro ECMO recirculation model. The early phase was defined as the period from SbO_2_ changes immediately after sweep gas off until S_post_O_2_ decreased from 99% to 95%, and the end phase as the final 5 s before S_post_O_2_ and S_pre_O_2_ converged. (**A**) Rf = 0%. (**B**) Rf = 50%. SbO_2_, blood oxygen saturation; ECMO, extracorporeal membrane oxygenation; Rf, recirculation fraction; S_pre_O_2_, inlet of the oxygenator; S_post_O_2_, outlet of the oxygenator

In the in vitro experiments, the hematocrit was 45% and remained constant throughout the procedures. **Figure 3** shows trends of S_post_O_2_ and S_pre_O_2_. The S_pre_O_2_ at Rf = 0% remained constant (**Fig. 3A**), whereas at Rf = 50%, it showed a time-dependent decreasing trend (**Fig. 3B**).

**Fig. 4:**
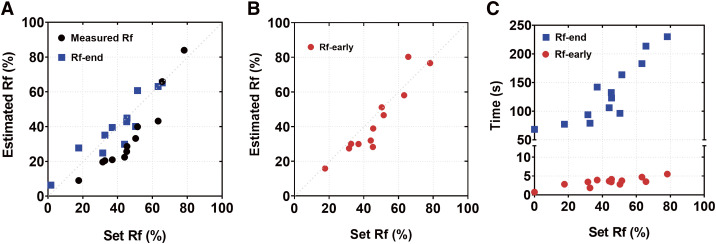
Recirculation estimations from the in vitro ECMO recirculation model. (**A**) Comparisons of Set Rf with Measured Rf and Rf-end. (**B**) Comparison between Set Rf and Rf-early. (**C**) Time required for Rf-early and Rf-end. Set Rf, predefined recirculation fraction set during the experiment; Rf Estimated in the early phase (Rf-early) and Rf Estimated in the end phase (Rf-end), predicted values obtained by our sweep-gas control method; Measured Rf, value calculated using directly measured venous saturation; ECMO, extracorporeal membrane oxygenation

The recirculation predicted by each method was compared with the recirculation measured using the flowmeter. The relative error was calculated using the following formula: |Set Rf – Estimated Rf (either Rf-early or Rf-end)|/Set Rf. The error values (mean ± standard deviation) were 12.5% ± 6.6% in the Measured Rf, 7.2% ± 7.2% in Rf-end (**Fig. 4A**), and 7.0% ± 5.2% in Rf-early (**Fig. 4B**), respectively. Rf-early was achieved significantly earlier than with the Measured Rf and Rf-end (**Fig. 4C**). The time required for Rf-early was 3.0 ± 1.0 s at Rf ≤50% and 4.1 ± 1.0 s at Rf >50%. That for Rf-end was 97.1 ± 25.8 s at Rf ≤50% and 177 ± 46.7 s at Rf> 50%.

**Fig. 5:**
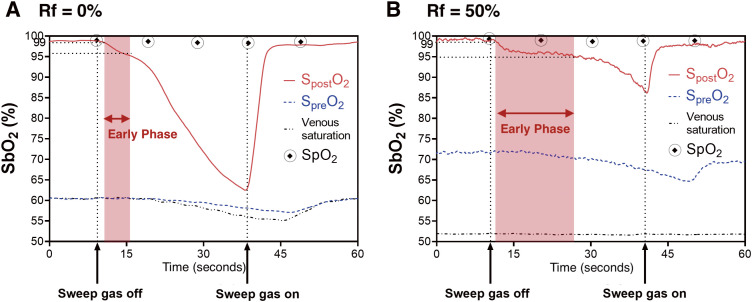
SbO_2_ changes in S_pre_O_2_, S_post_O_2,_ and venous saturation of the animal experiment. The early phase was defined as the period from SbO_2_ changes immediately after sweep gas off until S_post_O_2_ decreased from 99% to 95%. The sweep gas was resumed 30 s after being turned off. Venous saturation represents oxygen saturation measured at the drainage cannula. SpO_2_ was recorded at discrete time points and is shown as symbols. (**A**) Rf = 0%. (**B**) Rf = 50%. Rf, recirculation fraction; SbO_2_, blood oxygen saturation; S_pre_O_2_, inlet of the oxygenator; S_post_O_2_, outlet of the oxygenator

**Fig. 6:**
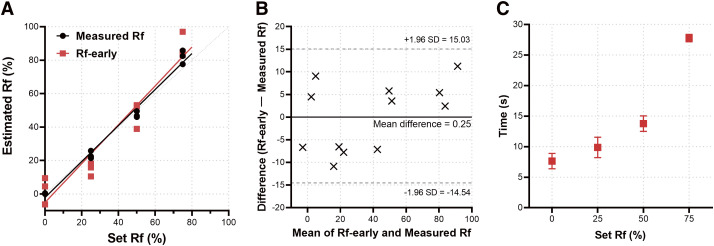
Recirculation estimations in the animal experiment. (**A**) Comparison of Set Rf with Measured Rf and Rf-early. Linear regression and ANCOVA comparing predicted and measured Rf values (Rf-early: *y* = 1.16*x* − 5.1, *R*^2^ = 0.93; Measured Rf: *y* = 1.08*x*− 2.1, *R*^2^ = 0.99). The dashed gray line indicates the line of identity (*y* = *x*). (**B**) Bland–Altman plot showing the mean bias (solid line) and 95% limits of agreement (dashed lines). (**C**) Time required for the Rf-early. Rf, recirculation fraction; ANCOVA, analysis of covariance

In the animal experiments, the hematocrit was 28% and remained stable throughout and systemic arterial pressure and heart rate were continuously monitored and remained stable throughout each measurement. Arterial oxygen saturation (SpO_2_) and venous oxygen saturation were also recorded during each measurement, including the period of sweep-gas cessation. Neither parameter showed a clinically significant decrease during the measurement window. S_pre_O_2_ and S_post_O_2_ showed similar trends to those in the in vitro experiments (**Fig. 5**). A time lag between S_pre_O_2_ and S_post_O_2_ was observed, caused by the length of the circuit. This lag was corrected by applying a time-shift to the S_pre_O_2_ signal. The delay time was calculated as the difference between the onset of the increase in S_post_O_2_ and the onset of the corresponding increase in S_pre_O_2_ after sweep-gas resumption. Rf-early values showed a positive correlation under the present experimental conditions with those derived from the Measured Rf (R^2^ = 0.93 vs. 0.99, respectively). Linear regression analysis demonstrated no significant difference in slope or intercept between the 2 methods (p >0.05). Bland–Altman analysis showed a mean bias of 0.25%, with limits of agreement of −14.5% to +15.0% (**Figs. 6A** and **6B**). The error values were 7.5% ± 4.0% at Rf ≤50% and 13.3% ± 6.2% at Rf = 75%; the prediction error at Rf = 75% was larger than that at Rf ≤50%. The time required for the Rf-early was less than 30 s in all experiments (**Fig. 6C**). Notably, the time required for Rf-early was 10.4 ± 2.8 s at Rf ≤50%.

## Discussion

We proposed a method to predict recirculation in ECMO by analyzing SbO_2_ trends at the pre- and post-membrane oxygenator sites and compared its performance with the Set Rf and Measured Rf following the same principle as the conventional SvO_2_ method. Rf-end using Eq. (3) demonstrated similar accuracy to the Measured Rf calculated from Eq. (2) using directly measured venous saturation. However, Rf-early in the range of S_post_O_2_ from 99% to 95% also showed comparable accuracy, and the time required for prediction was much faster than that required by the Measured Rf or Rf-end.

A key advantage of our proposed method over existing techniques is that recirculation can be estimated solely by manipulating the sweep-gas flow. This enables a simple bedside procedure and allows for easy integration into ECMO devices. Moreover, because the prediction time is short, near real-time estimation may be possible.

The accuracy of our method was demonstrated by a relative error of approximately 7% when the recirculation rate was below 50%. Inadequate oxygenation during VV ECMO can result from various factors. By enabling simple and real-time visualization of recirculation, our method may assist clinicians in distinguishing recirculation-related inefficiency from other hemodynamic or mechanical causes, thereby facilitating more targeted management and potentially improving circuit optimization.

Previous studies using UDT and computational fluid dynamics (CFD) analysis have shown that Rf generally remains below 50% but spans a wide range.^10,17,21,23)^ Higher recirculation levels are encountered in clinical practice and do not necessarily require immediate intervention if systemic oxygenation is maintained. At higher recirculation levels, the proposed method showed reduced absolute accuracy and longer prediction times, particularly at Rf = 75% in the animal experiments, which is expected because higher recirculation prolongs oxygen saturation equilibration. Nevertheless, despite increased absolute error, the method preserved sensitivity to changes in recirculation, with estimated values showing consistent directional trends in response to increasing Rf. This suggests that the proposed method may still be useful for detecting marked increases in recirculation and for monitoring temporal changes, even when precise absolute quantification becomes less reliable. Therefore, in high-Rf conditions, the current algorithm should be interpreted primarily as a screening or trend-monitoring tool rather than a definitive quantitative measurement. In clinical practice, such information may still be valuable for identifying conditions requiring further evaluation, such as cannula malposition, excessive pump flow, or unfavorable drainage-return interaction. Future refinements, including nonlinear calibration, Rf-range-specific correction factors, or confirmatory assessment using established reference methods, may improve quantitative accuracy in high-recirculation conditions.

Although transient sweep-gas cessation may raise safety concerns, it is important to note that, in the present study, both SpO_2_ and venous saturation remained stable throughout the measurement period, including during sweep-gas interruption (**Fig. 5**). These findings suggest that no acute deterioration in systemic oxygenation or oxygen supply–demand balance occurred during the short interruption period under the present experimental conditions.

However, these results should be interpreted with caution, as the experiments were conducted in animals with preserved lung function. In clinical settings, particularly in patients with severe acute respiratory distress syndrome who are highly dependent on ECMO gas exchange, even short interruptions may pose a risk. Therefore, clinical application would require strict safety monitoring, including continuous arterial oxygen saturation and hemodynamic assessment, and immediate resumption of sweep gas if instability occurs.

Equations (1)–(3) describe a linear relationship between SbO_2_ and the recirculation ratio, assuming the mixing of oxygenated and deoxygenated blood under quasi-steady conditions. This formulation is based on several simplifying assumptions, including stable venous oxygen saturation during the brief measurement window, linear mixing behavior, and operation within the near-linear region of the oxygen dissociation curve. This linearity holds under the condition that the oxygen dissociation curve remains in its steep, non-saturated region—typically below 99% SbO_2_.^24)^ However, at SbO_2_ levels exceeding 99%, the oxygen dissociation curve flattens, and changes in partial pressure of oxygen no longer translate to meaningful changes in SbO_2_. In our experiments, S_post_O_2_ showed minimal variation in this high-saturation range, making it difficult to apply Eq. (3) accurately. Therefore, the Rf-early was defined as the period in which S_post_O_2_ decreases from 99% to 95%. This range corresponds to the linear region of the oxygen dissociation curve and allows for valid application of the mixing equations. Although Eqs. (1)–(3) do not incorporate the oxygen transfer characteristics of the membrane, we observed that its performance remained stable throughout the Rf-early and Rf-end. It is likely that proportional changes in S_pre_O_2_ and S_post_O_2_ contributed to internal cancellation of membrane effects within the prediction formula.

This study had several limitations. First, a direct comparison with UDT, which is widely considered a reference method for recirculation assessment, was not performed in this study. Therefore, the external validity of the proposed method remains limited. The present results demonstrate internal consistency and feasibility under controlled conditions but do not establish equivalence to existing reference methods. Future studies should include direct comparison with UDT or other established techniques in clinical settings. Second, the number of animals in this study was limited to 3, which restricts the statistical robustness and generalizability of the findings. Although multiple recirculation conditions were tested in each animal, these repeated measurements do not substitute for biological replication. Therefore, the reported statistical parameters should be interpreted cautiously. Third, Eq. (3) does not explicitly account for dynamic changes in venous oxygen saturation. Although the model assumes quasi-steady conditions during the measurement window, this assumption may not hold in all physiological settings. It was difficult to mimic the clinical changes in venous saturation levels because the lung function of the pigs was normal. A change in venous saturation causes an error in Rf prediction. However, the Rf-early achieved the prediction within 15 s (**Fig. 6C**), which is significantly shorter than the systemic circulation time. The venous saturation would remain relatively stable during the measurement window, minimizing its impact on prediction accuracy. Fourth, in clinical situations, the recirculation could change depending on the patient’s respiratory and heart rate cycles. In particular, spontaneous respiratory efforts or stress-induced hyperventilation during sweep-gas interruption may alter ΔS_pre_O_2_ and ΔS_post_O_2_, potentially confounding recirculation estimation. This method was designed to obtain the average Rf during the sweep-gas off time, 30 s in this experiment, of the membrane. However, we have estimated a constant Rf in relation to time and have not been able to evaluate the accuracy when the Rf is dynamically changing. Future studies are needed to evaluate the robustness of the method in various clinical scenarios, including conditions with significant spontaneous breathing. Fifth, the experimental setup, including cannula placement, was designed to facilitate direct Rf measurement but does not fully replicate the clinical VV ECMO configuration. In addition, residual oxygen within the membrane oxygenator, non-instantaneous oxygen transfer dynamics, and circuit transit delay may influence the observed changes in oxygen saturation following sweep-gas interruption, leading to unintended oxygenation—a factor not considered in the analysis. The proposed method was designed as a rapid estimation tool based on early-phase changes rather than full equilibration and was evaluated using a single membrane oxygenator model; therefore, differences in retained oxygen volume, metabolic demand, and equilibration behavior across patients and oxygenator designs may affect post-oxygenator oxygen saturation changes during sweep-gas interruption. In clinical settings, reducing the sweep-gas oxygen fraction to accelerate post-oxygenator desaturation could induce transient hypoxemia and physiological stress, potentially altering cardiac output and confounding Rf estimation. Accordingly, future studies should evaluate oxygenator effects, validate the method using CFD, and compare its performance with UDT under clinical cannulation. In addition, automated sweep-gas control will be essential for clinical application.

## Conclusion

We proposed a novel method for estimating recirculation in the ECMO circuit using real-time optical SbO_2_ monitoring combined with transient sweep-gas control. The temporal change in SbO_2_ at the pre-oxygenator site during the decrease in post-oxygenator SbO_2_ from 99% to 95% was associated with the recirculation ratio. The method demonstrated promising accuracy under controlled conditions in both in vitro and animal experiments. As it requires no invasive procedures, this approach may provide a practical and rapid means of estimating recirculation at the bedside, although further validation is required before clinical application.

## Supplementary Material

Supplementary Material

## Declarations

### Data availability

The data that support the findings of this study are available from the corresponding author upon reasonable request.
