# Supplementary material for: A Novel Method for Predicting Recirculation by Sweep-Gas Control in Extracorporeal Membrane Oxygenation
Source: Ann Thorac Cardiovasc Surg. 2026 Jul 16;32(1):26-00027. doi: 10.5761/atcs.oa.26-00027 (PMC13377292; doi:10.5761/atcs.oa.26-00027)
Supplement: Supplementary Material [file atcs-32-1-26-00027-s001.pdf]

Derivation of the relationship between recirculation fraction and oxygen saturation changes

Recirculation fraction (Rf) is defined as:

$$Rf = \frac{Q_{rec}}{Q_{ec}} \qquad \text{(Eq.S1)}$$

Oxygen saturation at the pre-oxygenator site ($SpreO_2$) can be expressed as a mixture of recirculated blood ($SpostO_2$) and systemic venous blood (SvO_2):

$$S_{preO_2} = \frac{Q_{rec}}{Q_{ec}} S_{postO_2} + \frac{Q_{ec} - Q_{rec}}{Q_{ec}} SvO_2 \qquad \text{(Eq.S2)}$$

This equation assumes linear mixing of oxygen saturation under conditions where hemoglobin concentration is constant and oxygen content is approximately proportional to saturation within the relevant physiological range.

When sweep-gas is transiently discontinued for a short duration, oxygen transfer in the membrane oxygenator is reduced, resulting in changes in oxygen saturation at both pre- and post-oxygenator sites:

$$S_{preO_2} \rightarrow S'_{preO_2}$$
$$S_{postO_2} \rightarrow S'_{postO_2}$$

During this brief measurement window, SvO_2 is assumed to remain approximately constant. Applying Eq.S2 to both conditions (sweep-gas on and off) yields:

$$S'_{preO_2} = \frac{Q_{rec}}{Q_{ec}} S'_{postO_2} + \frac{Q_{ec} - Q_{rec}}{Q_{ec}} SvO_2 \qquad \text{(Eq.S3)}$$

Subtracting Eq.S3 from Eq.S2 eliminates the SvO_2 term:

$$S_{preO_2} - S'_{preO_2} = \frac{Q_{rec}}{Q_{ec}} (S_{postO_2} - S'_{postO_2}) \qquad \text{(Eq.S4)}$$

Defining:

$$\Delta S_{preO_2} = S_{preO_2} - S'_{preO_2}$$
$$\Delta S_{postO_2} = S_{postO_2} - S'_{postO_2}$$

we obtain:

$$\Delta S_{preO_2} = \frac{Q_{rec}}{Q_{ec}} \Delta S_{postO_2}$$

Thus:

$$Rf = \frac{\Delta S_{preO_2}}{\Delta S_{postO_2}} \qquad \text{(Eq.3)}$$

Assumptions and applicability

This derivation is based on the following assumptions:

1. Venous oxygen saturation (SvO_2) remains approximately constant during the short measurement period.
2. Hemoglobin concentration is constant.
3. Oxygen saturation operates within the quasi-linear region of the oxygen dissociation curve (approximately 95–99%).
4. Mixing between recirculated and systemic venous blood is homogeneous.
5. The measurement window is short relative to circuit transit time, allowing a quasi-steady approximation.

Potential deviations from these assumptions (e.g., rapid SvO_2 fluctuation, non-linear saturation behavior, or circuit transit delay) may introduce estimation error and are discussed in the main text. In particular, at very high oxygen saturation levels (approaching 100%), elevated PO_2 increases the contribution of dissolved oxygen, making oxygen content less proportional to saturation and potentially introducing estimation error. To minimize this effect, measurements were focused on the range where $SpostO_2$ decreases from 99% to 95%.
